# Optimising classification of Parkinson’s disease based on motor, olfactory, neuropsychiatric and sleep features

**DOI:** 10.1038/s41531-021-00226-2

**Published:** 2021-09-24

**Authors:** Jonathan P. Bestwick, Stephen D. Auger, Anette E. Schrag, Donald G. Grosset, Sofia Kanavou, Gavin Giovannoni, Andrew J. Lees, Jack Cuzick, Alastair J. Noyce

**Affiliations:** 1 Preventive Neurology Unit, Wolfson Institute of Population Health, Barts and the London School of Medicine and Dentistry, Queen Mary University of London, London, UK; 2 Department of Clinical and Movement Neuroscience, UCL Institute of Neurology, University College London, London, UK; 3 Department of Neurology, Institute of Neurological Sciences, Queen Elizabeth University Hospital, Glasgow, UK; 4 Population Health Sciences, University of Bristol, Bristol, UK; 5 Centre for Neuroscience, Surgery and Trauma, Blizard Institute, Barts and the London School of Medicine and Dentistry, Queen Mary University of London, London, UK

**Keywords:** Predictive markers, Risk factors

## Abstract

Olfactory loss, motor impairment, anxiety/depression, and REM-sleep behaviour disorder (RBD) are prodromal Parkinson’s disease (PD) features. PD risk prediction models typically dichotomize test results and apply likelihood ratios (LRs) to scores above and below cut-offs. We investigate whether LRs for specific test values could enhance classification between PD and controls. PD patient data on smell (UPSIT), possible RBD (RBD Screening Questionnaire), and anxiety/depression (LADS) were taken from the Tracking Parkinson’s study (*n* = 1046). For motor impairment (BRAIN test) in PD cases, published data were supplemented (*n* = 87). Control data (HADS for anxiety/depression) were taken from the PREDICT-PD pilot study (*n* = 1314). UPSIT, RBDSQ, and anxiety/depression data were analysed using logistic regression to determine which items were associated with PD. Gaussian distributions were fitted to BRAIN test scores. LRs were calculated from logistic regression models or score distributions. False-positive rates (FPRs) for specified detection rates (DRs) were calculated. Sixteen odours were associated with PD; LRs for this set ranged from 0.005 to 5511. Six RBDSQ and seven anxiety/depression questions were associated with PD; LRs ranged from 0.35 to 69 and from 0.002 to 402, respectively. BRAIN test LRs ranged from 0.16 to 1311. For a 70% DR, the FPR was 2.4% for the 16 odours, 4.6% for anxiety/depression, 16.0% for the BRAIN test, and 20.0% for the RBDSQ. Specific selections of (prodromal) PD marker features rather than dichotomized marker test results optimize PD classification. Such optimized classification models could improve the ability of algorithms to detect prodromal PD; however, prospective studies are needed to investigate their value for PD-prediction models.

## Introduction

Parkinson’s disease (PD) affects about 1% of individuals over the age of 60 years^1^. Clinical PD diagnosis is usually made late in the disease process and current treatments only relieve symptoms. Identifying earlier stages of PD may increase chances of slowing disease progression^2,3^. Accordingly, risk prediction models have been developed. In PREDICT-PD, a pilot study of 1323 individuals aged 60–80 years recruited from the general UK population^4^, the risk of PD was estimated based upon systematic review and meta-analysis of risk factors and early features^5^. Separately, the Movement Disorders Society (MDS) produced criteria for the diagnosis of prodromal PD^6,7^, a risk algorithm based upon primary-care presentations has also been described^8^, as well as risk algorithms based on clinical and genetic classification^9,10^. Most of these algorithms dichotomize exposure variables and risk factors, which in turn can lead to a loss of information if the underlying trait is continuous or discrete^11^.

In reporting the baseline and year 3 follow-up data from the PREDICT-PD pilot study, preliminary support for the validity and the value of the risk algorithm was assessed by comparing ‘intermediate outcome markers’ for PD between those at estimated highest and lowest risk. These intermediate markers comprised three of the strongest indicators of increased PD risk: olfactory loss, reduced finger-tapping speed, and possible rapid eye movement (REM)-sleep behaviour disorder (RBD)^4,12^. These markers, along with age, and anxiety and depression scores, represent the continuous data used in most prediction settings.

In the PREDICT-PD study, RBD was assessed subjectively using the RBD Screening Questionnaire (RBDSQ), with a score of ≥5 indicating possible RBD^13^. Olfactory loss was assessed objectively using the University of Pennsylvania Smell Identification Test (UPSIT), a 40-item ‘scratch-and-sniff’ smell test^14^. A score ≤ 15^th^ centile was used to indicate olfactory loss, which equated to an UPSIT score of ≤27^4,12^. Recently, members of our group used a data-driven approach to propose smaller subsets of the 40 items in the UPSIT, which could be used on a wider scale to predict olfactory loss^15^. Finger-tapping speed was used as an objective, quantitative motor marker with the Bradykinesia Akinesia Incoordination (BRAIN) test^16^. Users completed the BRAIN test online by alternately tapping the ‘S’ and ‘:’ keys on a keyboard as rapidly and accurately as possible in 30 s. The best two parameters generated are the kinesia score (KS, the total number of key taps) and the akinesia time (AT, the mean dwell time of keys in milliseconds). In year 3 of follow-up in PREDICT-PD, a KS score ≤44 (≤15th centile) signified reduced tapping speed^12^. In the PREDICT-PD algorithm, anxiety and depression contributed to risk estimation and was assessed using the Hospital Anxiety and Depression Scale (HADS)^17^ with scores ≥11, indicating moderate anxiety or depression.

Common to all PD-prediction models is the modification of the age-related risk of PD using the presence or absence of risk factors or protective factors. The MDS criteria do this using likelihood ratios based on results of prospective studies: e.g., the likelihood ratios for those with olfactory loss (denoted LR+) are 6.4 and 0.40 (denoted LR−) for those without; for possible RBD (based on the RBDSQ), the likelihood ratios (with and without) are 2.8 and 0.89, for abnormal quantitative motor testing the likelihood ratios (with and without) are 3.5 and 0.60, and for anxiety or depression the likelihood ratios (with and without) are 1.6 and 0.87^7^.

Here we investigate whether the use of likelihood ratios generated from the full range of test values or test items rather than using likelihood ratios for dichotomized test results optimizes classification of PD. Furthermore, we explored whether inclusion of objective measures (age, finger tapping, and smell) were more valuable than subjective traits (anxiety and depression, and possible RBD).

## Results

### Smell

**Fig. 1:**
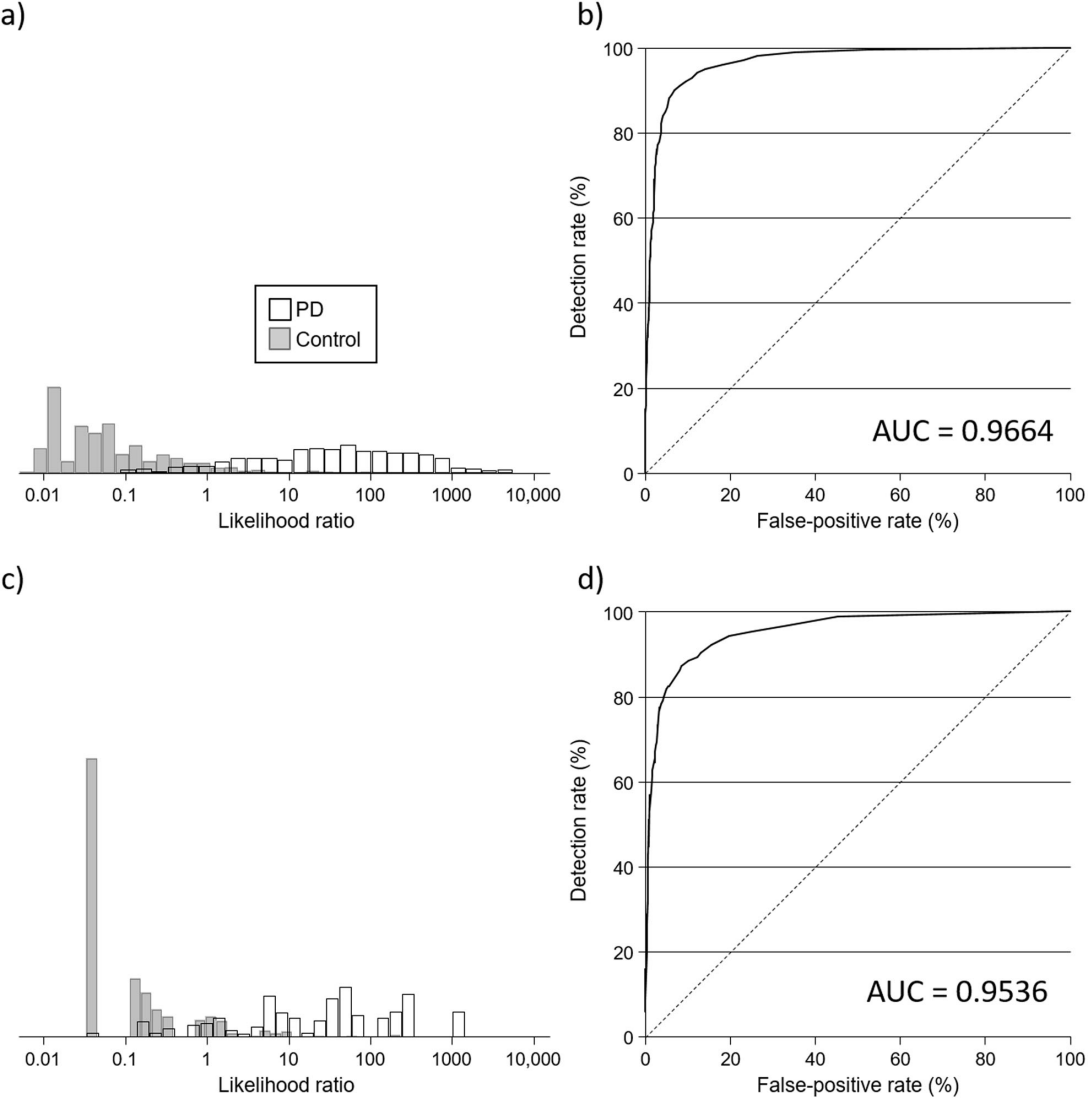
Classification of Parkinson’s Disease (PD) based on smell. **a** Distribution of likelihood ratios among PD cases and controls using the 16 odours of the University of Pennsylvania Smell Identification test (UPSIT) common to both the UK and US versions of the UPSIT identified as being associated with PD (see Table 1). **b** Observed detection rate according to false-positive rate (receiver operating characteristic [ROC] curve) for the 16 odours of the UPSIT identified as being associated with PD. **c** Distribution of likelihood ratios among PD cases and controls using the 6 odours common to both the UK and US versions of the UPSIT most strongly associated with PD (see Table 1). **d** ROC curve for the 6 odours of the UPSIT most strongly associated with PD. AUC, area under the receiver operating characteristic curve.

The median number of correctly identified odours (out of 40) was 18 among PD cases and 32 among controls (*p* < 0.001). Among the 32 odours common to both the UK and US versions of the UPSIT, correlation coefficients between pairs of odours were low; all <0.3, with 95% of correlations <0.2. Table 1 shows the results of the multivariate logistic regression analyses on items from the UPSIT. In a multivariate model, of the 32 odours, 16 were found to be statistically significantly associated with PD (Supplementary Table 1 shows univariate odds ratios for each of the 32 odours) with the remaining 16 odours not adding to the model. The table also shows the results for the six odours that were most strongly associated with PD. Figure 1 shows the distribution of likelihood ratios and receiver operating characteristic (ROC) curves for each set of odours. Based on the 16 odours, the median likelihood ratios for PD were 36 (95% confidence interval (95% CI) 29–44) and 0.05 (95% CI 0.04–0.06) in PD cases and controls, respectively (*p* < 0.001), and ranged from 0.009 to 5511 in PD cases (10th–90th centile 1.10–556) and from 0.005 to 515 in controls (10th–90th centile 0.02–0.68). For 50%, 60%, 70%, and 80% detection rates, false-positive rates were 1.1%, 1.9%, 2.4%, and 3.7%, respectively. Corresponding internally validated estimates were 1.4%, 2.2%, 2.7%, and 4.2%, respectively. The area under the ROC curve (AUC) was 0.97 and the internally validated AUC was 0.96. Using an UPSIT cut-off of ≤27, as was done when UPSIT scores were previously dichotomized^4,12^, the detection rate was 85% and the false-positive rate 14.9%. Using lower cut-offs of 23 and 25, the detection and false-positive rates were 84% and 6.2%, and 85% and 9.4% respectively; higher cut-offs yielded the same performance as with the cut-off of 27. For the same 14.9% false-positive rate for the cut-off of 27, the detection rate for the 16 odours was 10 percentage points higher (95% vs. 85%). Based on the 6 odours that were most strongly associated with PD in this analysis, the median likelihood ratios were 32 (95% CI 24–33) in PD cases and 0.03 (95% CI 0.03–0.03) in controls (*p* < 0.001), and ranged from 0.03 to 1429 in PD cases (10th–90th centile 0.72–305) and from 0.03 to 315 in controls (10th–90th centile 0.03–1.07), and corresponding false-positive rates for 50%, 60%, 70%, and 80% detection rates were 0.9%, 1.6%, 2.8%, and 4.4%, respectively. Corresponding internally validated estimates were 0.9%, 1.7%, 2.9%, and 4.4%, respectively. The AUC was 0.95 and the internally validated AUC was also 0.95. For the same 14.9% false-positive rate using an UPSIT cut-off of ≤27, the detection rate for the six odours was 92%.

**Table 1:** Results of multivariate logistic regression analyses of the odours common to the UK and US versions of the UPSIT, and the 6 odours most strongly associated with Parkinson’s disease.

Odour	Model including all significant odours			Model including 6 odours most strongly associated with PD			Likelihood ratio test statistics between nested models	
	Coefficient	OR (95% CI)	*p*-Value	Coefficient	OR (95% CI)	*p*-Value	*χ*^2^	*p*-Value
Gasoline	−1.492617	0.22 (0.15–0.34)	5.66 × 10^−12^	−1.952729	0.14 (0.1–0.21)	2.17 × 10^−24^	663.52	2.57 × 10^−146^
Soap	−1.956232	0.14 (0.10–0.21)	4.43 × 10^−23^	−2.232339	0.11 (0.08–0.15)	1.36 × 10^−34^	354.48	4.486 × 10^−79^
Watermelon	−1.436059	0.24 (0.15–0.37)	6.69 × 10^−11^	−1.859984	0.16 (0.11–0.23)	5.82 × 10^−21^	230.33	5.042 × 10^−52^
Lemon	−1.402295	0.25 (0.17–0.35)	1.40 × 10^−14^	−1.511823	0.22 (0.16–0.3)	6.21 × 10^−20^	131.11	2.337 × 10^−30^
Cinnamon	−1.174883	0.31 (0.21–0.45)	8.66 × 10^−10^	−1.544792	0.21 (0.15–0.3)	2.35 × 10^−18^	96.36	9.601 × 10^−23^
Natural gas	−1.084457	0.34 (0.21–0.54)	6.15 × 10^−6^	−1.561454	0.21 (0.14–0.32)	7.06 × 10^−13^	55.57	9.03 × 10^−14^
Rose	−0.8652221	0.42 (0.28–0.63)	2.71 × 10^−5^				38.76	4.79 × 10^−10^
Paint thinner	−0.8597572	0.42 (0.29–0.61)	6.03 × 10^−6^				28.29	1.05 × 10^−7^
Pineapple	−0.7759867	0.46 (0.30–0.71)	0.0004				20.42	6.21 × 10^−6^
Banana	−0.6495947	0.52 (0.35–0.77)	0.0011				13.07	0.0003
Cedar	−0.5584326	0.57 (0.39–0.84)	0.0048				10.25	0.0014
Cherry	−0.5074133	0.60 (0.41–0.88)	0.0087				7.12	0.0076
Strawberry	0.6116164	1.84 (1.21–2.81)	0.0045				7.00	0.0082
Coconut	−0.4896007	0.61 (0.41–0.91)	0.0143				5.87	0.0154
Menthol	−0.6320504	0.53 (0.32–0.88)	0.0148				4.22	0.0400
Mint	0.5117308	1.67 (1.08–2.57)	0.0209				5.48	0.0192
Constant	8.66404	5790.88 (2480.46–13519.39)	3.09 × 10^−89^	7.313897	1501.02 (754.9–2984.58)	1.33 × 10^−96^

Supplementary Table 2 shows results for the multivariate model for the six odours previously identified as being predictive of olfactory loss^15,18^. Supplementary Fig. 1a shows the distribution of likelihood ratios in PD cases and controls, and Fig. 1b shows the ROC curve based on these six odours. The median likelihood ratios were 6.0 (95% CI 5.5–7.1) in PD cases and 0.14 (95% CI 0.14–0.14) in controls (*p* < 0.001), and ranged from 0.14 to 112 in PD cases (10th–90th centile 0.55–51) and from 0.14 to 195 in controls (10th–90th centile 0.14–1.91; 0.14 is the LR assigned to someone that correctly identified all 6 odours; 59% of controls did so). For 50%, 60%, 70%, and 80% detection rates, false-positive rates were 4.1%, 5.3%, 8.5%, and 14.7%, respectively (i.e., poorer performance than for the six odours that were most strongly associated with PD in this analysis). Corresponding internally validated estimates were 4.2%, 5.4%, 8.7%, and 15.0%, respectively. The AUC was 0.88 and the internally validated AUC was also 0.88.

For the alternative approach examining the total UPSIT scores, there was a small but significant decrease in UPSIT scores with increasing age (0.61 per 5 years of age, 95% CI 0.31–0.92; *p* < 0.001) and males overall had lower scores than females (1.26 points lower, 95% CI 0.66–1.87; *p* < 0.001). The regression equations for males and females are given in Eqs. (1) and (2), respectively:

1 $${{{\mathrm{UPSIT}}}}\,{{{\mathrm{score}}}} = 39.59 - 0.1221 \times {{{\mathrm{age}}}}$$

2 $${{{\mathrm{UPSIT}}}}\,{{{\mathrm{score}}}} = 40.86 - 0.1221 \times {{{\mathrm{age}}}}$$

Using these equations to convert to delta values gave the distribution of scores shown in Supplementary Fig. 2a. In both PD cases and controls, there was evidence of a mixture of two Gaussian distributions (*p* < 0.001 for both, indicating mixtures of two Gaussian distributions fit the data better than a single Gaussian distribution in PD cases and a single Gaussian distribution in controls).

In neither PD cases nor controls was there evidence of more than two underlying distributions. The means, SDs, and mixing proportions are given in the Supplementary Material. Supplementary Fig. 2b shows the likelihood ratio according to UPSIT score multiple of the median (MoM) values. Risk reversal occurred below UPSIT delta values of −23.71 and above 4.15, and truncation limits were applied at these points. The median likelihood ratio in PD cases was 10.2 (95% CI 8.9–11.3) and in controls was 0.12 (95% CI 0.12–0.13) (*p* < 0.001) with a range in likelihood ratios from 0.07 to 45 (10th–90th centile 0.32–37.6 in PD cases, 0.07–1.67 in controls). Supplementary Fig. 2c shows an ROC curve for the UPSIT delta values. For observed detection rates of 50%, 60%, 70%, and 80%, the false-positive rates were 2.5%, 3.6%, 4.7%, and 8.7%, respectively, higher than the false-positive rates derived from the results of the logistic regression analyses. The AUC was 0.91, lower than the AUC values based on the logistic regression analyses. For the same 14.9% false-positive rate using an UPSIT cut-off of ≤27, the detection rate for the mixture distributions was 85%.

### Possible REM-sleep behaviour disorder

**Fig. 2:**
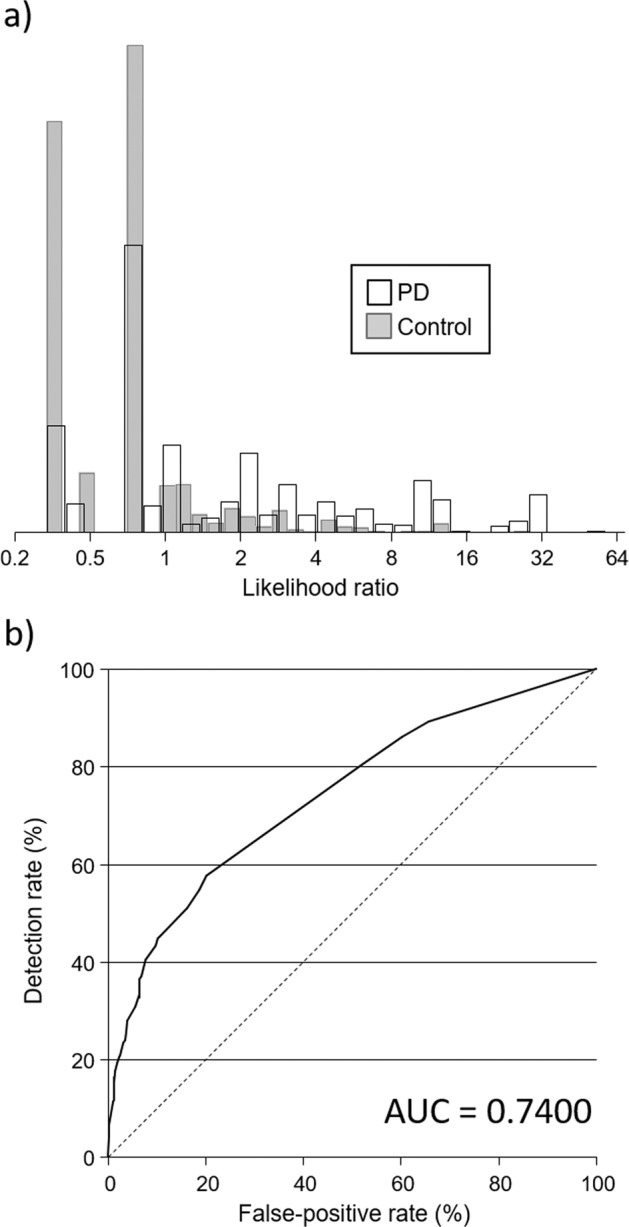
Classification of Parkinson’s Disease (PD) based on the REM-sleep behaviour Screening Questionnaire (RBDSQ). **a** Distribution of likelihood ratios among PD cases and controls using the 6 questions of the RBDSQ identified as being associated with PD (see Table 2). **b** The observed detection rate according to false-positive rate (receiver operating characteristic curve) for the 6 questions of the RBDSQ identified as being associated with PD. AUC, area under the receiver operating characteristic curve.

Table 2 shows the results of the multivariate logistic regression analyses on items of the RBDSQ. Six of the 12 questions were significantly associated with being a PD case (Supplementary Table 3 shows univariate odds ratios for each question of the RBDSQ). Figure 2a shows the distribution of likelihood ratios and Fig. 2b an ROC curve based on the results in Table 2. The median likelihood ratio was 1.14 (95% CI 1.00–1.14) in PD cases and 0.80 (95% CI 0.70–0.79) in controls (*p* < 0.001), and the range was 0.35–69 in PD cases (10th–90th centile 0.35–11) and 0.35–29 (10th–90th centile 0.35–1.6) in controls. For a 50% detection rate, the false-positive rate was 16%; for detection rates of 60%, 70%, and 80%, the false-positive rate was constant at 20%. Corresponding internally validated estimates were 12.2%, 20.4%, 20.3%, and 13.2%, respectively. The AUC was 0.74 and the internally validated AUC was 0.73. Using a cut-off of ≥5 as is typically done when RBDSQ scores are dichotomized, the detection rate was 35% and the false-positive rate 14.1%. For the same 14.1% false-positive rate, the detection rate for the 16 odours was 51%.

**Table 2:** Results of multivariate logistic regression analyses of the questions that make up the REM-sleep behaviour Screening Questionnaire (RBDSQ).

RBDSQ question	Coefficient	OR (95% CI)	*p*-Value	Likelihood ratio test statistics between nested models	
				*χ*^2^	*p*-Value
5. It thereby happened that I (almost) hurt my bed partner or myself	1.566722	4.79 (3.26 –7.04)	1.44 × 10^−15^	23.15	2.88 × 10^−52^
3. The dream contents mostly match my nocturnal behaviour	1.044969	2.84 (2.14–3.78)	5.38 × 10^−13^	73.62	9.47 × 10^−18^
1. I sometimes have very vivid dreams	−0.872100	0.42 (0.34–0.51)	5.89 × 10^−17^	49.64	1.84 × 10^−12^
6. I have or had any of the following phenomena during my dreams:					
6.1 Speaking, shouting, laughing very loudly	0.7731019	2.17 (1.71–2.74)	9.31 × 10^−11^	57.56	3.28 × 10^−14^
6.4 Things that fell down around the bed, e.g., bedside lamp, book, glasses	0.7478248	2.11 (1.38–3.24)	0.0006	13.99	0.0002
4. I know that my arms or legs move when I sleep	0.3166089	1.37 (1.09–1.73)	0.0076	7.03	0.0080
Constant	−0.6268203	0.53 (0.46–0.62)	1.50 × 10^−17^

### Anxiety and depression

**Fig. 3:**
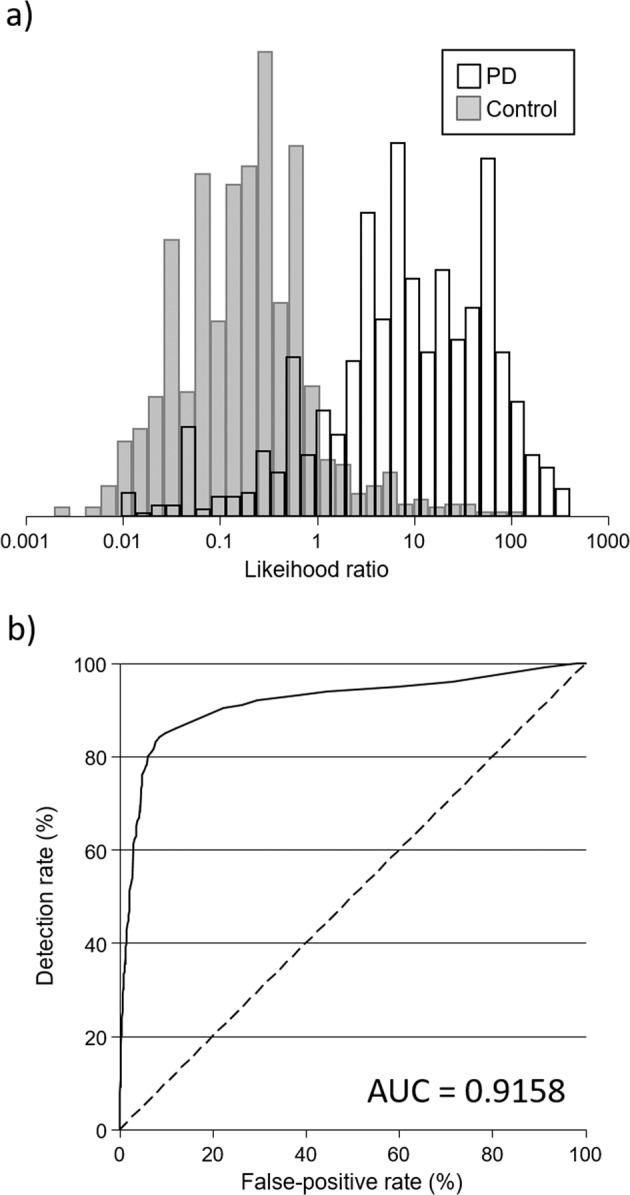
Classification of Parkinson’s Disease (PD) based on anxiety and depression scales. **a** Distribution of likelihood ratios among PD cases and controls using the seven questions common to both the Hospital and Leeds Anxiety and Depression Scales (HADS and LADS), respectively, used in PREDICT-PD and Tracking Parkinson’s, which were associated with PD (see Table 3). **b** The observed detection rate according to false-positive rate (receiver operating characteristic curve) for the seven questions common to the HADS and LADS identified as being associated with PD. AUC, area under the receiver operating characteristic curve.

Table 3 shows the results of the multivariate logistic regression analyses on items common to both the HADS and Leeds Anxiety and Depression Scale (LADS). Seven of the nine questions were significantly associated with being a PD case in a multivariate model with the question “I feel as if I am slowed down” being most strongly associated with PD (OR = 10.3, 95% CI 8.43–12.58) (Supplementary Table 4 shows univariate odds ratios for each question common to both the HADS and LADS). Figure 3a shows the distribution of likelihood ratios and Fig. 3b an ROC curve based on the results in Table 3. The median likelihood ratio was 8.2 (95% CI 6.2–10.2) in PD cases and 0.19 (95% CI 0.18–0.21) in controls (*p* < 0.001), and the range was 0.010–402 in PD cases (10th–90th centile 0.57–77) and 0.002–132 in controls (10th–90th centile 0.03–0.9). For 50%, 60%, 70%, and 80% detection rates, false-positive rates were 2.1%, 2.9%, 4.6%, and 6.1%, respectively. Corresponding internally validated estimates were 2.2%, 2.9%, 4.7%, and 6.2%, respectively. The AUC was 0.92 and the internally validated AUC was 0.91. As the question “I feel as if I am slowed down” may be subject to bias given that slowness of movement is a main symptom of PD and cases in the analysis were recently diagnosed, a post hoc sensitivity analysis was performed excluding this question. With this question excluded, false-positive rates were notably higher (19%, 33%, 53%, and 58% for 50%, 60%, 70%, and 80% detection rates, respectively) and the AUC substantially lower (0.71). The range of risks were also much narrower; 0.16–56 in PD cases (10th–90th centile 0.47–6.26) and 0.16–13.5 in controls (10th–90th centile 0.35–1.74).

**Table 3:** Results of multivariate logistic regression analyses for the items common to both the Hospital and Leeds Anxiety and Depression Scales (HADS and LADS) used in PREDICT-PD and Tracking Parkinson’s.

PREDICT-PD (HADS) question	Tracking Parkinson’s (modified LADS) question	Coefficient	OR (95% CI)	*p*-Value	Likelihood ratio test statistics between nested models	
					*χ*^2^	*p*-Value
8. I feel as if I am slowed down	13. I feel as if I have slowed down	2.3354	10.3 (8.43–12.58)	1.61 × 10^−115^	1271.87	1.47 × 10^−278^
3. I get a sort of frightened feeling like something awful is about to happen	2. I get very frightened or have panic feelings for apparently no reason at all	−0.5103821	0.55 (0.44–0.68)	2.87 × 10^−8^	59.69	1.11 × 10^−14^
11. I feel restless as if I have to be on the move	11. I am restless and can’t keep still	0.5628471	0.58 (0.49–0.69)	1.58 × 10^−9^	28.32	1.03 × 10^−7^
6. I feel cheerful	3. I feel miserable and sad^a^	0.2458486	1.85 (1.48–2.32)	9.47 × 10^−8^	30.80	2.87 × 10^−8^
2. I still enjoy the things I used to enjoy	10. I still enjoy the things I used to	−0.1423261	0.61 (0.49–0.75)	6.00 × 10^−6^	15.34	0.0001
12. I look forward with enjoyment to things	5. I have lost interest in things^a^	−0.5956851	1.39 (1.11–1.74)	0.004	6.75	0.009
13. I get sudden feelings of panic	8. I feel scared or frightened	−0.3123743	0.76 (0.60–0.97)	0.030	4.70	0.030
Constant		−3.250492	0.04 (0.03–0.05)	5.35 × 10^−102^		

^a^Scale reversed.

### Quantitative motor impairment

For the BRAIN test scores, KS followed a Gaussian distribution, and AT did after log-transformation. There was a small but significant decrease in KS scores with increasing age (−1.02 per 5 years of age, 95% CI −0.31 to −1.72; *p* = 0.005) and females overall had higher scores (1.49 higher, 95% CI 0.10 to 2.87; *p* = 0.035). The regression equations for males and females are given in Eqs. (3) and (4), respectively.

3 $${{{\mathrm{KS}}}} = 66.10 - 0.2030 \times {{{\mathrm{age}}}}$$

4 $${{{\mathrm{KS}}}} = 67.59 - 0.2030 \times {{{\mathrm{age}}}}$$

There was a small but significant increase in log (natural) AT scores with increasing age (0.04 per 5 years of age, 95% CI 0.01–0.06; *p* = 0.001) and females overall had higher scores (0.08 higher, 95% CI 0.04–0.12; *p* < 0.001). The regression equations for males and females are given in Eqs. (5) and (6), respectively.

5 $$\ln \left( {{{{\mathrm{AT}}}}} \right) = 4.126 + 0.006932 \times {{{\mathrm{age}}}}$$

6 $$\ln \left( {{{{\mathrm{AT}}}}} \right) = 4.211 + 0.006932 \times {{{\mathrm{age}}}}$$

**Fig. 4:**
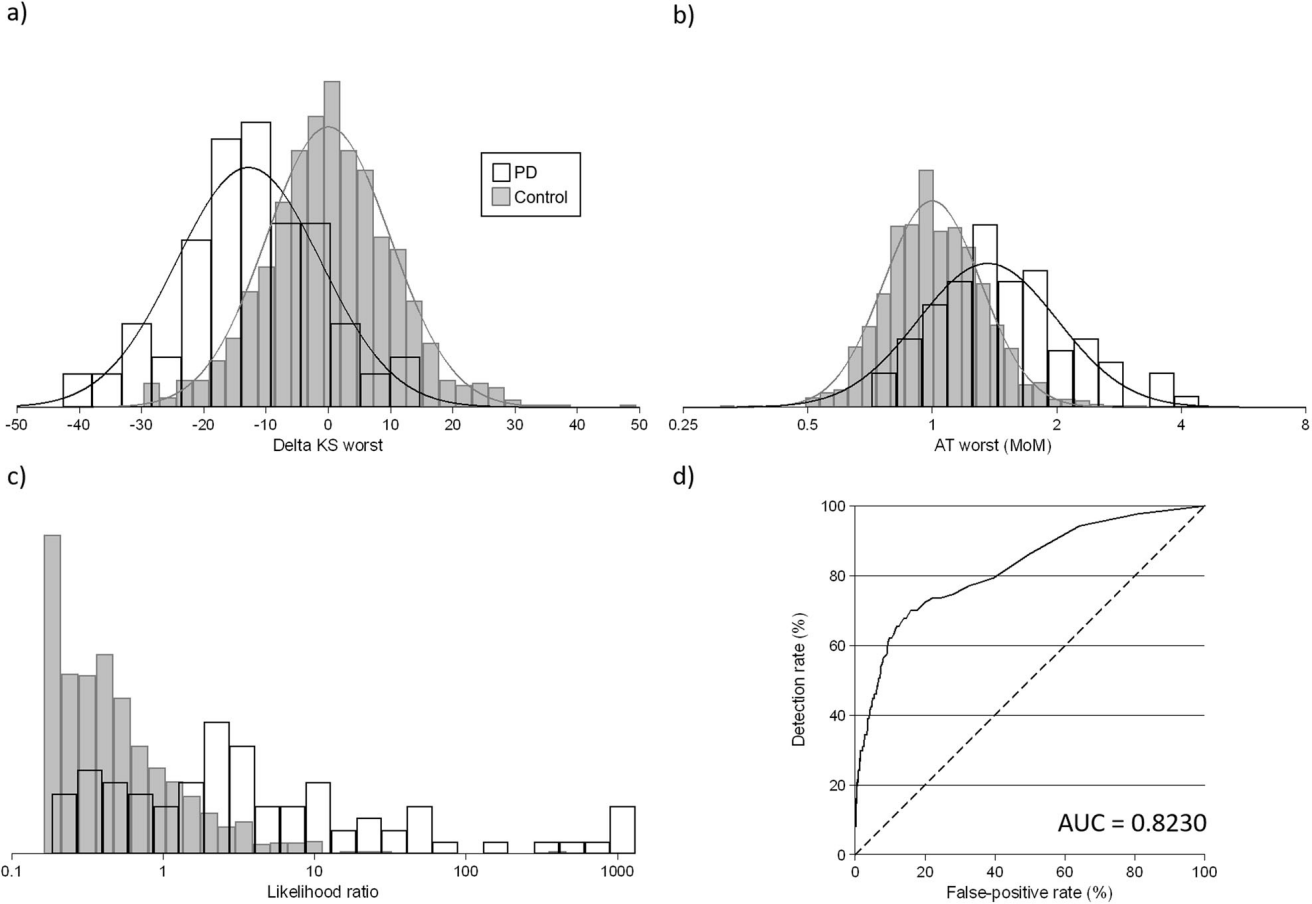
Classification of Parkinson’s Disease (PD) based on the Bradykinesia Akinesia Incoordination (BRAIN) test. **a** Distributions of kinesia score (difference from the median [delta] KS) in PD cases and controls. **b** Distributions of akinesia time (multiple of the median [MoM] AT) in PD cases and controls. **c** Distributions of likelihood ratios based on the multivariate Gaussian distributions of KS and AT. **d** The observed detection rate according to false-positive rate (receiver operating characteristic curve) based on the multivariate Gaussian distributions of KS and AT. AUC, area under the receiver operating characteristic curve.

After transforming values into delta values for KS and MoM values for AT using the above regression equations, mean KS values were 12.8 points lower in PD cases than in controls, and AT values 36% higher (both *p* < 0.001). Supplementary Fig. 3 shows probability plots for delta KS values (3a) and AT MoM values (3b), with AT MoM values plotted on a log scale. Delta KS values start to deviate, or the data were sparse, below −30 and above 10, and for AT below 0.5 MoM and above 3.0 MoM. These values were therefore used as truncation limits. However, the point of risk reversal for AT was at 0.75, so values less than this were truncated. All distributions were reasonably Gaussian as indicated by the points roughly falling on straight lines. Figure 4 shows the distribution of delta KS (4a) and AT MoM (4b) values in PD cases and controls. KS had the best discrimination between PD cases and controls. Supplementary Table 5 shows the parameters (means, SDs, correlation coefficients, truncation limits) in PD cases and controls. Figure 4c shows the distributions of likelihood ratios in PD cases and controls. The median likelihood ratio in PD cases was 2.73 (95% CI 1.99–3.95) and in controls was 0.39 (95% CI 0.37–0.42) (*p* < 0.001) with a range in likelihood ratios from 0.18 to 1311 in PD cases (10th–90th centile 0.35–81) and 0.16–458 in controls (10th–90th centile 0.18–1.9). Figure 4d shows an ROC curve for the combination of delta KS and AT MoM values. For 50%, 60%, 70%, and 80% detection rates, false-positive rates were 6.7%, 9.3%, 16.0%, and 40%, respectively. Corresponding internally validated estimates were the same to one decimal place. The AUC was 0.82 and the internally validated AUC was the same to two decimal places. Using a KS cut-off of ≤44 as was done when the scores were previously dichotomized, the detection rate was 62% and the false-positive rate 16.5%. For the same 16.5% false-positive rate, the detection rate using the new approach was 62%.

### Combining likelihood ratios generated from different tests

Pairwise correlation coefficients between likelihood ratios generated from each test and scale are shown separately for PD cases and controls in Supplementary Table 6 (among PD cases, it was not possible to calculate correlations between likelihood ratios from the BRAIN test and any of likelihood ratios generated from UPSIT, RBDSQ, or anxiety, and depression tests and scales). Correlation coefficients were between −0.03 and 0.07, and despite some just reaching statistical significance, this indicates the tests are largely independent and likelihood ratios can be multiplied together in risk estimation. Supplementary Table 7 shows screening performance when likelihood ratios based on the RBDSQ, and/or anxiety and depression are combined with likelihood ratios for the UPSIT (using the 16 or 6 most discriminating odours), limited to PD cases (*n* = 835) and controls (*n* = 887) with complete data. The addition of likelihood ratios for the RBDSQ yielded only a small improvement in screening performance. For example, using the 16 odours that discriminated between PD cases and controls, for an 80% detection rate the false-positive rate decreased from 3.6% to 3.0%, but at a 90% detection rate the false-positive rate increased from 6.4% to 6.7%. The AUC was, however, statistically significantly higher at 0.971 with and 0.967 without the RBDSQ (*p* = 0.006). The addition of likelihood ratios for anxiety and depression to likelihood ratios for the 16 items of the UPSIT did yield an improvement in screening performance (false-positive rates of 1.5% and 3.9% for 80% and 90% detection rates respectively; AUC = 0.986, *p* < 0.001 compared with the AUC for the 16 items of the UPSIT [0.967]) but when the question “I feel as if I am slowed” was excluded the improvement was trivial (false-positive rates of 3.2% and 6.2% for 80% and 90% detection rates, respectively).

### Sensitivity analyses

Supplementary Table 8 shows the sensitivity analysis results of the multivariate conditional logistic regression analyses on items from the UPSIT in which controls were matched to PD cases on the basis of age category and gender. Of the 32 odours, 11 were found to be statistically significantly associated with PD. Ten of these odours were found to be associated with PD in the full dataset and odds ratios are similar. Only onion was found to be associated with PD in the matched dataset but not in the full dataset. The table also shows the results for the six odours that were most strongly associated with PD, which were the same six odours that were identified using the full dataset.

Supplementary Table 9 shows the sensitivity analysis results of the multivariate conditional logistic regression analyses on items from the UPSIT in which PD cases with dementia or cognitive impairment were excluded. Of the 32 odours, 14 were found to be statistically significantly associated with PD. Twelve of these odours were found to be associated with PD in the full dataset and odds ratios are similar. Chocolate and motor oil were found to be associated with PD in the dataset excluding PD cases with dementia or cognitive impairment but not in the full dataset. The table also shows the results for the six odours that were most strongly associated with PD, which were the same six odours that were identified using the full dataset.

## Discussion

Our results show that maximizing information on markers for PD that are either continuous or discrete substantially extends the range of likelihood ratios than is achieved by dichotomization and can improve screening performance in terms of an increase in the detection rate for the same false-positive rate observed using dichotomized test results. In comparison to dichotomous likelihood ratios for olfactory performance of 6.4 for those with olfactory loss vs. 0.40 for those with normosmia^7^, olfactory likelihood ratios presented here ranged from 0.07 to 45 based on the full range of olfactory performance (using the total score from the 40-item UPSIT) and ranged from 0.009 to about 5500 using the logistic regression approach that identified 16 odours, which were significantly associated with PD. Similarly, possible RBD likelihood ratios ranged from 0.35 to 69, which compares favourably to respective likelihood ratios of 0.89 and 2.8 for those without or with possible RBD previously reported^7^. For anxiety and depression, likelihood ratios ranged from 0.002 to 402 compared to likelihood ratios of 0.87 and 1.6 for those without and with at least moderate anxiety or depression, although the range was narrower after excluding the question “I feel as if I am slowed down” (0.16 to 56). Finally, likelihood ratios for finger-tapping speed based on two BRAIN test parameters ranged from 0.16 to about 1300, whereas previous dichotomized values were 0.60 and 3.5 for those without and with abnormal quantitative motor testing, respectively^7^. Although the range of likelihood ratios is large, it does occur that likelihood ratios can be small for some PD cases and large for some controls, even though overall there is an improvement in performance. Any revision to an algorithm will ultimately reclassify some people incorrectly where they may have previously been categorized correctly, but for the majority the reclassification will be correct.

The practical use of likelihood ratios that are specific to an individual’s test or scale responses is no different to the use of positive or negative likelihood ratios, which depend on a cut-off used to dichotomize scores. The positive likelihood ratio is the detection rate divided by the false-positive rate (or sensitivity divided by the complement of the specificity). The resulting range of possible likelihood ratios is large in comparison with the likelihood ratios in the MDS research criteria for prodromal PD^6,7^. The likelihood ratios in the MDS research criteria are based on prospective studies and incident PD, whereas this study is cross-sectional with recently diagnosed PD cases, which represents a limitation and direct comparison of likelihood ratios is not possible. Although the range of likelihood ratios presented here is large, conceptually it is reasonable, e.g., that an individual that was only able to identify a third of odours be assigned a likelihood ratio greater than an individual who was able to identify half of the odours, whereas when dichotomizing they would both be assigned the same likelihood ratio. Similarly, it is reasonable that an individual who could identify all odours be assigned a lower likelihood ratio than an individual who could identify 75% of odours. Although the likelihood ratios presented here may appear extreme, in other areas of medicine, risk prediction algorithms can yield far more extreme likelihood ratios. For example, with the commonly used ‘Combined test’ (one ultrasound marker, two serum markers, and maternal age) in prenatal screening for Down syndrome, likelihood ratios of >1,000,000 are possible^19^.

The logistic regression approach to UPSIT provided more information than examining total UPSIT scores and offers potential improvement compared to simply dichotomizing scores. There are a number of different tests of smell on the market, such as the Sniffin’ Sticks and conversion of scores from this test to the full 40-item UPSIT has been described^20^. For researchers using alternative smell tests, conversion could be performed and likelihood ratios for total UPSIT scores applied (using Supplementary Fig. 2b). Otherwise, a logistic regression approach could be used to develop a model to calculate likelihood ratios using different smell tests provided there are sufficient data. We presented two six-item odour tests, one from previous work that selected items on their ability to predict olfactory loss in healthy controls^15,18^ and a new selection based on their ability to differentiate PD cases from controls in the current analysis. The 6-item test from the current analysis had similar performance to the test using 16 of the 32 odours common to both the UK and US UPSIT. The six-item test based on previous work performed less well, but this test may have an advantage in that it was primarily designed to test for olfactory loss, in which case it could be more generalizable to diseases other than PD and has been externally validated in a dataset independent to the one used to derive it^18^. In any case, either six-item test could offer substantial cost savings over routinely using the full UPSIT when administered on a large scale.

Other studies have also have investigated subsets of the UPSIT or other smell tests^21–24^. The most comparable to our study was by Morley et al.^24^ who examined 12 items of the UPSIT and, using a logistic regression approach, found an AUC of 0.80 and, using a cut-off of 8/12 correctly identified odours, a sensitivity of 82% and specificity of 68%^24^. They found, however, that the discriminatory power was not the same in two independent cohorts (for the 40 UPSIT items discriminatory power was higher in the independent cohorts) but this was judged solely by the AUC, which is not recommended^25,26^. A preferred method is comparison of sensitivity for given reasonable (i.e., higher) levels of specificity and/or specificity for given reasonable (higher) levels of sensitivity^26^. Where subsets of smell tests have been studied, there has been a lack of uniformity in the odours that are associated with PD with one of the possible reasons being because of cultural or other differences between populations^24^. In this study, this is somewhat mitigated by the fact that the PD cases completed the UK version of the UPSIT and the controls of the US version, so we only examined the 32 odours common to both versions.

Although there is an advantage in dichotomizing variables to clinically easily interpretable thresholds for categorizing cases, in other areas of medicine such as a patient having hypertension or not, dichotomizing variables comes at the cost of loss of information^11^ and is avoided in other areas of medical risk-assessment calculations (e.g., using actual blood pressure readings in cardiovascular disease prediction rather than dichotomizing at a fixed value as hypertensive or not). Using individual questions for tests or scales such as the UPSIT, RBDSQ, or HADS/LADS, or using information on the exact scores of tests such as the BRAIN test maximizes information but is likely to be of most use for objective measures such as the UPSIT and BRAIN test. Although we were unable to combine likelihood ratios for the BRAIN test with those from the other tests, combining likelihood ratios for the RBDSQ with those from the UPSIT did not materially affect screening performance, and when combining UPSIT and HADS/LADS likelihood ratios, there was an improvement in screening performance (Supplementary Table 7), but not with the exclusion of the question ‘I feel as if I am slowed’. This question particularly is likely subject to bias given that slowness of movement is a main symptom of PD and cases in the analysis were recently diagnosed; in those who get a PD diagnosis in the future, the odds ratio is likely to be smaller than the value of 10.3 observed in this study. Similarly, the question ‘Worrying thoughts constantly go through my mind’ could resemble PD-related worries rather than indicate depression. The lack of improvement in screening performance with the addition of likelihood ratios based on multivariate logistic regression models for the RBDSQ and HADS/LADS could be a reflection of the subjective nature of these tests; as such, there is unlikely to be much advantage in calculating likelihood ratios based on individual questions from these scales over dichotomizing. Further work based on prospective data and incident PD would be needed before considering the use of likelihood ratios based on specific answers to questions of the RBDSQ and HADS/LADS.

In this study, we have used an approach similar to that which has been used for many years in prenatal screening for Down syndrome, as well as more recently in prenatal screening for trisomy 18, trisomy 13, and preeclampsia. In the same way as MoM values take account of natural changes in ultrasound and serum markers with gestational age in prenatal screening for Down syndrome and similar conditions^19,27^, our delta and MoM values take account of normal changes in smell loss and tapping parameters with age, and also between males and females. Researchers wishing to use delta or MoM values could generate these from their own data in the same way as done here, using regression analysis among those without PD, or in a cohort study by regression analysis in all participants. Emerging blood or other biomarkers for PD would also benefit from the use of delta or MoM values instead of mass units, with the added advantage of accounting for of systematic differences between different assays and laboratories by calculating delta or MoM values based on local data. The use of multivariate Gaussian distributions also allows for a modular approach, i.e., adding new markers to risk prediction models as they are discovered, without needing data on each marker to be measured in the same participants, albeit under the assumption of independence with established markers.

A weakness of this study is that the estimates of screening performance are based on cross-sectional marker distributions in those with diagnosed PD. In practice, such risk estimation will take place before diagnosis, so screening performance estimates in a ‘healthy population’ are likely to be lower than those presented here. To mitigate this as much as possible, we used the earliest possible data collected in the Tracking Parkinson’s study, such that we used the baseline assessments of RBD, and anxiety and depression, which were performed on average 1.4 years after PD diagnosis and the UPSIT was performed on average 6.7 months after baseline (on average, 1.9 years since PD diagnosis). Further, olfactory deficits have often been shown to be independent of PD disease duration and disease stage^21,28^. Our logistic regression analysis of the UPSIT did not adjust for age and gender, because in algorithms such as the MDS research criteria for prodromal Parkinson’s and the PREDICT-PD risk algorithm, the age-specific risk is modified by the likelihood ratios according to gender^6,7,12^, so to include age and gender in the multivariate logistic regression models would double count age and gender in the algorithm. Nevertheless, the sensitivity analysis in which PD cases and controls were matched on age and gender produced similar results, including the same six odours in the six-item test, which is not surprising given that even though females and younger participants scored statistically significantly higher on the UPSIT, the effect of age and gender was small. Similarly, we were unable to adjust for cognitive deficit, because this was defined using Montreal Cognitive Assessment scores in PD cases, but equivalent data were not available in controls. A large population-based study found that after adjustment for age, sex, education, and depressive symptoms, higher smell test scores were significantly associated with better verbal abilities and semantic memory, better ability to learn new verbal information, verbal memory, and delayed free recall, attention or cognitive processing speed, and executive function^29^. Therefore, given the cross-sectional case–control nature of this study, we cannot exclude bias arising from cognitive deficits. However, in our sensitivity analysis where PD cases with dementia or cognitive impairment were excluded, reassuringly the same six odours were associated with PD, with similar odds ratios to those using the full dataset.

Ideally, the approaches used in this study would be assessed and likelihood ratios generated using prospective data, but until cohorts that include the assessments examined in this study mature so that they include a large enough number of individuals diagnosed with PD with sufficient follow-up, such as the PREDICT-PD cohort, this study represents a starting point and, until prospective data with enough PD cases is available, the LRs presented here can be used to calculate PD risk in a research framework. However, to what the degree the classification models of the present study can be translated and used for PD-prediction models will require further methodological investigation. We have, however examined the likelihood ratios presented here for smell, tapping speed, RBD and anxiety and depression together with age and other factors, such as smoking status and family history of PD, to determine by how much the spread of risk increases in the PREDICT-PD cohort, and how risk estimates are associated with PD diagnosis in the limited number that have been diagnosed so far in this cohort^30^. In this study, we examined each test or scale separately to avoid a large reduction in the number of PD cases and controls that would have resulted were we to have jointly examined the tests and scales where possible. However, pairwise correlations between likelihood ratios obtained from the separate tests in both PD cases and controls were very small, indicating that the tests are largely independent. In practice, therefore, it is possible to multiply likelihood ratios from the separate tests in estimating PD risk. It is recognized that estimates of performance of a predictive model are often overestimated when determined on the sample from which the model is derived due to overfitting^31^, but our internally validated estimates of performance were very similar. We used the bootstrap method for internal validation, which provides a level of optimism of the observed AUC and detection rates for specified false-positive rates and false-positive rates for specified detection rates based on all data, and is a more efficient method of internal validation than splitting the dataset into training and validation sets, with the latter method being shown to be overly pessimistic^31,32^. Despite our internally validated estimates of the performance of the tests being similar to those based on the original data, showing good internal validation, the approach used here also needs to be externally validated in independent datasets and ideally assessed using prospective data with incident PD as the outcome. This will also determine whether the individual items of the UPSIT, RBDSQ, and depression and anxiety questions that were identified as being associated with PD can be replicated. A further weakness of this study is that data on PD cases came from a different source to that of controls, and for assessing anxiety and depression, different but overlapping scales were used. Although the age profiles were similar between PD cases and controls, because we limited to those aged 60–80 years, we cannot exclude the presence of residual confounding, reinforcing the need for external validation.

In summary, this study shows that maximizing information on continuous and discrete markers for PD has potential, by providing more precise risk estimates, to improve the ability of algorithms to detect PD and this study provides the methods for incorporating this approach into other algorithms. This approach needs to be validated in independent prospective datasets and the translation of the association models and LRs to valid prediction models is needed.

## Methods

### Data sources

In this cross-sectional case–control study, control data came from individuals in the PREDICT-PD pilot study who had not been diagnosed with PD during follow-up at year 6 (mean age 67 years, 62% female); participants had completed the UPSIT (*n* = 887) and/or BRAIN test (*n* = 1071), and/or RBDSQ (*n* = 1314) and/or HADS (*n* = 1314). Full details of the study were previously published^4^. Data from cases with PD were derived from several sources: the assessment of UPSIT and RBDSQ scores, and anxiety and depression were taken from baseline data of those aged between 60 and 80 years in the Tracking Parkinson’s study (young-onset PD cases were excluded), a multicentre prospective longitudinal epidemiological and biomarker study of PD (*n* = 1046, mean age 69 years, 47% female; RBDSQ scores available on *n* = 983; anxiety and depression data available on *n* = 872)^33^. For the BRAIN test, 59 PD cases came from previously published data^34^, supplemented with unpublished data for a total of *n* = 87 PD cases. BRAIN tests were performed ‘off’ treatment in PD cases.

### Calculation of likelihood ratios

For the full 40-item UPSIT, total scores were adjusted by performing a median regression of UPSIT scores against age and gender among controls, then subtracting all participants’ UPSIT scores from their expected score from the regression equations (UPSIT delta values). Previous work, based on the University of Pennsylvania 12-item Brief Smell Identification Test, showed that 9/49 PD cases had normal olfactory function and 12/52 controls had abnormal olfactory function^21^; therefore, we expected there would be a mixture of distributions of total UPSIT scores in both PD cases and controls. To investigate whether there was a mixture of Gaussian distributions among PD cases and controls, finite mixture models with more than one Gaussian component were fitted. From the final distributions, likelihood ratios according to UPSIT delta values were calculated as the height of the modelled distribution (density) in PD cases divided by the height of the modelled distribution in controls, with truncation limits applied to avoid risk reversal^35^ (see Supplementary Material). In addition to using the full 40-item UPSIT score, we also performed logistic regression to determine which odours were associated with PD. A forward stepwise procedure was used for this analysis (with a 0.05 significance level for entry into the model), using the 32 odours that were common to both the UK and US version of the UPSIT (the PREDICT-PD pilot study used the US version, whereas the Tracking Parkinson’s study used the UK version). We also explored the performance of a subset of the six odours most strongly associated with PD identified from the forward stepwise procedure. We further examined the results of a six-item smell test, which was based on the four odours that discriminated most between those with and without olfactory loss from previous work, plus a further two odours that most discriminated between PD and controls (menthol, clove, orange, onion, coconut, and cherry)^15,18^. Using the logistic regression approach, likelihood ratios were calculated by calculating the log odds based on the final multivariate logistic regression models, exponentiating and then dividing by 932/887; the number of PD cases divided by the number of controls in the analysis.

For the BRAIN test, results for the worst of KS and AT scores from each hand were adjusted by performing a median regression of scores against age and gender among controls, then either subtracting (for KS) or dividing (for AT) all participants’ scores by the expected score from the regression equations. Fits of adjusted BRAIN test scores to Gaussian distributions were assessed by inspection of probability plots. The points at which data started to deviate from a Gaussian distribution were used as truncation limits, to avoid extreme KS or AT values having an unwanted impact on the combined likelihood ratio. Where there were any points of risk reversal within the limit, the point of risk reversal was used as a truncation limit. The means, SDs, and correlation coefficients between the two parameters were calculated to define a bivariate Gaussian distribution in PD cases and controls. To avoid the influence of outliers, the median was used as the mean and robust SDs were calculated as the 90th centile minus the 10th centile divided by 2.563 (i.e., number of SDs between the 10th and 90th centiles of a Gaussian distribution). Likelihood ratios according to adjusted KS and AT values were calculated as the height of the bivariate Gaussian distribution in PD cases, divided by the height of the bivariate Gaussian distribution in controls (see Supplementary Material for formula).

For total RBDSQ scores, the final question that scores 1 if a person was diagnosed with a disease of the nervous system was not included, given that all PD cases would score 1. As with analyses on UPSIT scores, multivariate logistic regression using a forward stepwise procedure was used to determine which questions in the RBDSQ were associated with PD. Likelihood ratios were calculated by calculating the log odds based on the fitted multivariate logistic regression model, exponentiating and then dividing by (875/1314).

For assessment of anxiety and depression, the PREDICT-PD pilot study used the HADS, whereas the Tracking Parkinson’s study used the LADS^36^ with the addition of two questions from HADS. The LADS consists of six questions related to anxiety and six to depression; four of the anxiety questions and three of the depression questions matched questions in the HADS (of the three depression questions, two were the converse of the questions in the HADS, so reversing the scores for these two questions equated them). Therefore, there were nine questions common to both the PREDICT-PD and Tracking Parkinson’s cohorts that could be used in the analysis; multivariate logistic regression using a forward stepwise procedure was used to determine which of these were associated with PD, with the scores for each question, which ranged from 0 to 3 (Likert-type scales) treated as linear, to preserve the ordinal nature of responses and to minimize degrees of freedom. Likelihood ratios were calculated by calculating the log odds based on the fitted multivariate logistic regression model, exponentiating and then dividing by (872/1314).

For each marker and approach, the performance in predicting PD was estimated as false-positive rates for specified detection rates. The AUC was also calculated. Internal validation of the specified models was performed using the bootstrapping method^31,32^. Briefly, for the fitted logistic regression models using all data, a new model was fitted on a bootstrap sample, and that model tested on the bootstrap sample and on the original data, with the AUC and false-positive rates for specified detection rates calculated. This process was repeated 1000 times. The average difference in the AUC and false-positive rates for specified detection rates provided estimates of the optimism of the performance of the models fitted on all data. The estimates of optimism were then subtracted from the performance measures to estimate the internally validated performance. Similar bootstrap estimates of performance to the observed performance using the original full data indicate good internal validation. For the BRAIN test, a similar method was used but was based on fitting bivariate Gaussian distributions to bootstrap samples. In this analysis, setting truncation limits based on where the distributions deviated from a Gaussian distribution cannot be automated, so were the same as those based on all data, unless they were set at the point(s) of risk reversal. However, in practice, truncation limits have little impact on detection or false-positive rates or the AUC.

Sensitivity analyses were performed for the logistic regression approach to the UPSIT in which (i) a reduced dataset was used where controls were matched to PD cases by age (in 3-year categories) and gender, to determine whether this impacted the multivariate models given that younger people and females have been shown to perform better on the UPSIT, and (ii) a reduced dataset in which PD cases with dementia or cognitive impairment were excluded.

Spearman’s rank correlation was used to determine whether likelihood ratios generated from the different tests were independent. Statistical significance was set at 5% and all analyses were performed using Stata version 16 (StataCorp, College Station, Texas).

### Ethical approval

The PREDICT-PD study was approved by Central London Research Committee 3 (reference number 10/ H0716/85). Seventy-two sites in the United Kingdom providing secondary care treatment for PD patients as part of the UK National Health Service (and in selected sites, their linked academic institutions) are participating in the Tracking Parkinson’s study, with multicentre ethics committee and local research and development department approvals. All PREDICT-PD participants provided informed consent via the PREDICT-PD website. All Tracking Parkinson’s study participants provided written informed consent at the time of recruitment. Participants whose data were used, who were not part of the PREDICT-PD or Tracking Parkinson’s study, provided written informed consent. BRAIN test data used in this study that was not part of the PREDICT-PD or Tracking Parkinson’s studies were from patients recruited from the Movement Disorder clinic at the Royal London Hospital, approved by the Queen Square Research Ethics Committee (reference number 09/H0716/48) or the East London PD Project, approved by the Southwest Bristol Ethics Committee (reference number 18/SW/0255), and for both, participants provided written informed consent.

### Reporting summary

Further information on research design is available in the Nature Research Reporting Summary linked to this article.

## Supplementary information

Supplementary Information

Reporting Summary

## Data Availability

Applications for PREDICT-PD data are reviewed by the PREDICT-PD steering committee. Applications for Tracking Parkinson’s data access are reviewed by the Data & Biosample Access committee. This study used Tracking Parkinson’s (PRoBaND) dataset, version 1.0, 20190131. Other data used in this study is available upon reasonable request from the corresponding author, with approval from all authors.

## Code availability

All analyses were performed using Stata version 15 (StataCorp, College Station, Texas). Codes for data analysis in this study are available upon request.
