# Supplementary material for: Optimising classification of Parkinson’s disease based on motor, olfactory, neuropsychiatric and sleep features
Source: NPJ Parkinsons Dis. 2021 Sep 24;7:87. doi: 10.1038/s41531-021-00226-2 (PMC8463675; doi:10.1038/s41531-021-00226-2)

Optimising classification of Parkinson's disease based on motor, olfactory, neuropsychiatric and sleep features: Supplementary Material

Calculation of likelihood ratios for UPSIT delta scores and BRAIN test kinesia score and akinesia time

Likelihood ratios for total UPSIT delta scores based on a mixture of Gaussian distributions among PD patients and controls are calculated using the following formula:

$$LR = \frac{\sum_{i=1}^{n_{PD}} \left\{ p_{PD,i} \times \left(\frac{1}{\sigma_{PD,i}\sqrt{2\pi}} e^{-\frac{1}{2}\left(\frac{Delta-\mu_{PD,i}}{\sigma_{PD,i}}\right)^2} \right) \right\}}{\sum_{j=1}^{n_C} \left\{ p_{C,j} \times \left(\frac{1}{\sigma_{C,j}\sqrt{2\pi}} e^{-\frac{1}{2}\left(\frac{Delta-\mu_{C,j}}{\sigma_{C,j}}\right)^2} \right) \right\}}$$

where n_{PD} is the number of underlying Gaussian distributions in PD cases, and for i=1 to n_{PD}, $p_{PD,i}$ is the mixing proportion, $\mu_{PD,i}$ is the mean and $\sigma_{PD,i}$ the standard deviation for distribution i. Similarly n_C is the number of underlying Gaussian distributions in controls, and for i=1 to n_C, $p_{C,i}$ is the mixing proportion, $\mu_{PD,i}$ is the mean and $\sigma_{C,i}$ the standard deviation for distribution i.

To avoid the phenomena of risk reversal, which occurs when the standard deviation of a screening marker in an affected population is greater than that in an unaffected population, truncation limits were applied so that the likelihood ratio is a monotonically increasing function of UPSIT delta values.[20]

In PD patients the mixing proportions were 72% ($p_{PD,1}$) following a distribution with mean -15.57 ($\mu_{PD,1}$) and standard deviation 4.17 ($\sigma_{PD,1}$) and 28% ($p_{PD,2}$) folllowing a distribution with mean -4.51 ($\mu_{PD,2}$) and standard deviation 4.35 ($\sigma_{PD,2}$). In unaffected controls, the mixing proportions were 17% ($p_{C,1}$) folllowing a distribution with mean 7.07 ($\mu_{C,1}$) and standard deviation 5.97 ($\sigma_{C,1}$) and 83% ($p_{C,2}$) folllowing a distribution with mean 0.50 ($\mu_{C,2}$) and standard deviation 2.81 ($\sigma_{C,2}$).

Likelihood ratios according to adjusted KS and AT values are calculated using the following formula

$$LR = \frac{\frac{1}{(2\pi)^{3/2}|\Sigma_{PD}|^{1/2}} e^{\left(-\frac{1}{2}(x-\mu_{PD})^T \Sigma_{PD}{}^{-1}(x-\mu_{PD})\right)}}{\frac{1}{(2\pi)^{3/2}|\Sigma_C|^{1/2}} \mathrm{e}^{\left(-\frac{1}{2}(x-\mu_C)^T \Sigma_C{}^{-1}(x-\mu_C)\right)}}$$

Where Σ_{PD} is the covariance matrix in PD patients, $|\Sigma_{PD}|$ is the determinant of the covariance matrix in PD patients, μ_{PD} is the vector of the means of KS, AT and IS in PD patients, Σ_C is the covariance matrix in controls, $|\Sigma_C|$ is the determinant of the covariance matrix in controls, μ_C is the 1×2 vector of the means of KS MoM values and ln(AT MoM values) in controls, and x is the 1×2 vector of MoM values (KS MoM, ln(AT MoM)); T stands for transpose.

Supplementary Table 1: Univariate odds ratios for Parkinson's disease for the 32 common items on the US and UK versions of the Univeristy of Pennsylvania Smell Identification Test

Odour	OR (95% CI)	p-value
Pizza	0.20 (0.16 to 0.24)	6.82×10^{-55}
Bubblegum	0.24 (0.19 to 0.29)	1.38×10^{-44}
Menthol	0.07 (0.05 to 0.10)	6.16×10^{-58}
Cherry	0.15 (0.12 to 0.18)	4.26×10^{-68}
Motor oil	0.20 (0.17 to 0.25)	3.39×10^{-52}
Mint	0.24 (0.20 to 0.30)	4.46×10^{-39}
Banana	0.19 (0.15 to 0.24)	3.98×10^{-50}
Clove	0.08 (0.06 to 0.11)	1.16×10^{-67}
Leather	0.12 (0.09 to 0.17)	6.06×10^{-39}
Coconut	0.13 (0.10 to 0.16)	1.03×10^{-71}
Onion	0.17 (0.13 to 0.24)	2.49×10^{-27}
Cinnamon	0.11 (0.09 to 0.14)	7.84×10^{-78}
Gasoline	0.05 (0.04 to 0.07)	7.39×10^{-99}
Strawberry	0.34 (0.28 to 0.42)	8.84×10^{-26}
Cedar	0.23 (0.19 to 0.29)	7.09×10^{-44}
Chocolate	0.37 (0.30 to 0.46)	1.24×10^{-19}
Lilac	0.14 (0.10 to 0.19)	6.40×10^{-40}
Turpentine	0.51 (0.42 to 0.63)	1.21×10^{-10}
Peach	0.13 (0.10 to 0.16)	1.90×10^{-49}
Pineapple	0.10 (0.07 to 0.13)	7.11×10^{-69}
Lime	0.79 (0.66 to 0.96)	0.0182
Orange	0.14 (0.11 to 0.18)	2.41×10^{-55}
Watermelon	0.06 (0.04 to 0.08)	3.98×10^{-85}
Paint thinner	0.16 (0.13 to 0.20)	1.13×10^{-64}
Grass	0.65 (0.54 to 0.78)	6.90×10^{-6}
Smoke	0.11 (0.08 to 0.16)	1.06×10^{-33}
Pineapple	0.21 (0.17 to 0.26)	4.30×10^{-43}
Lemon	0.12 (0.09 to 0.14)	1.23×10^{-87}
Soap	0.08 (0.06 to 0.10)	3.62×10^{-87}
Natural gas	0.07 (0.05 to 0.09)	1.97×10^{-68}
Rose	0.09 (0.07 to 0.12)	1.44×10^{-79}
Peanut	0.15 (0.12 to 0.20)	4.33×10^{-47}

Supplementary Table 2: Results of multivariate logistic regression analyses of the four odours that discriminated most between those with and without hyposmia, plus a further two odours that most discriminated between Parkinson's disease and controls from previous work[1,2]

	Model based on previously identified odours			Likelihood ratio test statistics between nested models	
Odour	Coefficient	OR (95% CI)	p-value	χ^2	p-value
Coconut	-1.342963	0.26 (0.2 to 0.34)	1.46×10^{-21}	383.69	1.96×10^{-85}
Cherry	-1.330007	0.26 (0.2 to 0.34)	3.07×10^{-23}	447.60	2.40×10^{-99}
Clove	-1.422969	0.24 (0.17 to 0.33)	1.63×10^{-17}	345.69	3.68×10^{-77}
Menthol	-1.594804	0.2 (0.14 to 0.29)	1.20×10^{-17}	226.32	3.78×10^{-51}
Orange	-0.9652192	0.38 (0.28 to 0.52)	6.56×10^{-10}	162.71	2.90×10^{-37}
Onion	-0.554275	0.57 (0.38 to 0.86)	0.0076	124.20	7.60×10^{-29}
Constant	5.32388	205.18 (117.7 to 357.68)	1.19×10^{-78}		

[1]Joseph T, Auger SD, Peress L, et al. Screening performance of abbreviated versions of the UPSIT smell test. *J Neurol*, **226**, 1897-1906 (2019)
[2]Auger SD, Kanavou S, Lawton M, et al. Testing shortened versions of smell tests to screen for hyposmia in Parkinson's disease. *Mov Disord Clin Pract*, **21**, 7, 394-398 (2020)

Supplementary Table 3: Univariate odds ratios for Parkinson's disease (PD) for the items in the REM sleep behaviour disorder screening questionnaire

RBDSQ question	OR (95% CI)	p-value
1. I sometimes have very vivid dreams	0.76 (0.64 to 0.90)	0.0017
2. My dreams frequently have an aggressive or action-packed content	1.97 (1.59 to 2.45)	5.11×10^{-10}
3. The dream contents mostly match my nocturnal behaviour	4.28 (3.34 to 5.49)	1.44×10^{-30}
4. I know that my arms or legs more when I sleep	2.86 (2.36 to 3.47)	7.05×10^{-27}
5. It thereby happened that I (almost) hurt my bed partner or myself	9.57 (6.81 to 13.45)	1.38×10^{-38}
6. I have or had any of the following phenomena during my dreams:	3.20 (2.64 to 3.88)	3.90×10^{-32}
6.1 Speaking, shouting, laughing very loudly	2.90 (2.36 to 3.55)	1.71×10^{-24}
6.2 Sudden limb movements, "fights"	4.34 (3.08 to 6.11)	4.00×10^{-17}
6.3 Gestures, complex movements that are useless during sleep, e.g., to wave, to salute, to frighten mosquitoes, falls off the bed	5.76 (3.98 to 8.32)	1.37×10^{-20}
6.4 Things that fell down around the bed, e.g. bedside lap, book, glasses	2.82 (2.23 to 3.55)	2.53×10^{-18}
7. It happens that my movements awake me	0.91 (0.76 to 1.08)	0.2822
8. After awakening I mostly remember the content of my dreams well	1.11 (0.93 to 1.33)	0.2343
9. My sleep is frequently disturbed		

Supplementary Table 4: Univariate odds ratios for Parkinson's disease (PD) for the items common to both the Hospital and Leeds Anxiety and Depression Scales (HADS and LADS)

PREDICT-PD (HADS) question	Tracking Parkinson's (modified LADS) question	OR (95% CI)	p-value
Anxiety			
3. I get a sort of frightened feeling like something awful is about to happen	2. I get very frightened or have panic feelings for apparently no reason at all	0.88 (0.79 to 0.99)	0.036
5. Worrying thoughts go through my mind	14. Worrying thoughts constantly go through my mind	1.49 (1.34 to 1.65)	4.03×10^{-14}
9. I get a sort of frightened feeling like 'butterflies in the stomach'	6. I get palpitations, or sensations of 'butterflies' in my stomach or chest	1.18 (1.04 to 1.33)	0.009
11. I feel restless as if I have to be on the move	11. I am restless and can't keep still	0.88 (0.79 to 0.97)	0.012
13. I get sudden feelings of panic	8. I feel scared or frightened	1.17 (1.03 to 1.33)	0.016
Depression			
2. I still enjoy the things I used to enjoy	10. I still enjoy the things I used to	1.47 (1.29 to 1.68)	5.10×10^{-9}
6. I feel cheerful	3. I feel miserable and sad[1]	2.33 (2.05 to 2.65)	1.10×10^{-37}
8. I feel as if I am slowed down	13. I feel as if I have slowed down	9.02 (7.6 to 10.72)	4.55×10^{-138}
12. I look forward with enjoyment to things	5. I have lost interest in things[1]	2.42 (2.13 to 2.75)	4.40×10^{-43}

[1]Scale reversed

Supplementary Table 5: Marker parameters for the BRAIN test kinesia score (delta KS) and akinesia time (multiple of the median [MoM] AT) among Parkinson's disease (PD) patients and controls.

	**PD**	**Control**
KS Delta values		
Mean	-12.80	0
Standard deviation	11.62	9.94
Truncation limits	-30 to 10	
AT MoM values		
Mean ($\log_e$)	0.3087	0
Standard deviation ($\log_e$)	0.3876	0.2701
Truncation limits	0.747 to 3.0	
Correlation coefficients between KS Delta values and AT MoM values	-0.5626	-0.3487

Supplementary Table 6: Correlation coefficients (r) between likelihood ratios (LRs) calculated from each test or scale among Parkinson's disease (PD) patients and controls

	**PD**		**Controls**	
Correlation between LRs for:-	r	p-value	r	p-value
UPSIT (16 items) & RBDSQ	0.0686	0.042	0.0333	0.322
UPSIT (16 items) & HADS/LADS	0.0692	0.041	0.0384	0.254
UPSIT (16 items) & BRAIN-test	-	-	-0.0211	0.566
UPSIT (6 items) & RBDSQ	0.0699	0.039	0.0245	0.467
UPSIT (6 items) & HADS/LADS	0.0429	0.205	0.0289	0.391
UPSIT (6 items) & BRAIN-test	-	-	0.0152	0.679
RBDSQ & HADS/LADS	-0.0289	0.294	-0.0289	0.294
RBDSQ & BRAIN-test	-	-	0.0029	0.925
HADS/LADS & BRAIN-test	-	-	0.0703	0.021

UPSIT, Univeristy of Pennsylvania Smell Identification Test; RBDSQ, REM sleep behaviour disorder screening questionnaire; HADS, Hospital Anxiety and Depression Scale; LADS, Leeds Anxiety and Depression Scale; BRAIN-test, Bradykinesia Akinesia INcorodination test.

Supplementary Table 7: Screening performance of the 16- and 6-items of the Univeristy of Pennsylvania Smell Identification Test (UPSIT) that were associated with PD, and with the addition of items from the REM sleep behaviour disorder screening questionnaire (RBDSQ), and from the Hospital/Leeds Anxiety and Depression Scales (HADS/LADS) that were associated with PD in the PD patients (n=835) and controls (n=887) with complete data for the three tests.

	False-positive rate for detection rate of:-					
	50%	60%	70%	80%	90%	AUC
UPSIT (16 items)	1.1	1.9	2.3	3.6	6.4	0.967
UPSIT (16 items) & RBDSQ	0.9	1.0	1.9	3.0	6.7	0.971
UPSIT (16 items) & HADS/LADS	0.1	0.3	0.3	1.5	3.9	0.986
UPSIT (16 items) & HADS/LADS[1]	0.9	1.2	2.0	3.2	6.2	0.970
UPSIT (16 items) & RBDSQ & HADS/LADS	0.2	0.2	0.5	0.9	2.9	0.986
UPSIT (16 items) & RBDSQ & HADS/LADS[1]	0.5	0.9	1.6	2.6	5.0	0.974
UPSIT (6 items)	0.9	1.7	2.8	4.2	13.2	0.954
UPSIT (6 items) & RBDSQ	0.9	1.1	2.4	3.8	9.7	0.967
UPSIT (6 items) & HADS/LADS	0.0	0.3	0.3	1.4	4.1	0.983
UPSIT (6 items) & HADS/LADS[1]	0.9	1.2	2.4	3.0	12.7	0.939
UPSIT (6 items) & RBDSQ & HADS/LADS	0.2	0.2	0.5	1.0	4.1	0.984
UPSIT (6 items) & RBDSQ & HADS/LADS[1]	0.6	1.0	1.5	3.0	13.6	0.963

AUC, area under the receiver operation characteristic curve.

[1] Excluding the question “I feel as if I am slowed”

Supplementary Table 8: Results of conditional multivariate logistic regression analyses of the odours common to the UK and US versions of the UPSIT, and the 6 odours most strongly associated with Parkinson's disease in age and gender matched data

	Model including all significant odours			Model including 6 odours most strongly associated with PD		
Odour	Coefficient	OR (95% CI)	p-value	Coefficient	OR (95% CI)	p-value
Gasoline	-1.44206	0.24 (0.12 to 0.46)	2.42×10^{-5}	-1.609182	0.20 (0.11 to 0.36)	3.83×10^{-8}
Soap	-2.18692	0.11 (0.06 to 0.22)	4.42×10^{-11}	-2.252089	0.11 (0.06 to 0.19)	3.23×10^{-14}
Watermelon	-1.82095	0.16 (0.08 to 0.32)	1.48×10^{-7}	-1.841385	0.16 (0.09 to 0.29)	3.35×10^{-9}
Lemon	-1.31478	0.27 (0.15 to 0.47)	5.71×10^{-6}	-1.526365	0.22 (0.13 to 0.36)	2.34×10^{-9}
Cinnamon	-1.18593	0.31 (0.17 to 0.56)	1.34×10^{-4}	-1.524661	0.22 (0.13 to 0.38)	6.72×10^{-8}
Natural gas	-1.61344	0.20 (0.09 to 0.44)	5.92×10^{-5}	-1.694505	0.18 (0.09 to 0.36)	1.11×10^{-6}
Cherry	-0.89760	0.41 (0.22 to 0.74)	0.003			
Rose	-1.09199	0.34 (0.17 to 0.65)	0.001			
Banana	-0.91279	0.4 (0.23 to 0.71)	0.002			
Onion	1.22592	3.41 (1.31 to 8.84)	0.012			
Pineapple	-0.78406	0.46 (0.23 to 0.9)	0.023			

Supplementary Table 9: Results of conditional multivariate logistic regression analyses of the odours common to the UK and US versions of the UPSIT, and the 6 odours most strongly associated with Parkinson's disease (excluding patients with dementia or cognitive impairment)

	Model including all significant odours			Model including 6 odours most strongly associated with PD		
Odour	Coefficient	OR (95% CI)	p-value	Coefficient	OR (95% CI)	p-value
Gasoline	-1.677869	0.19 (0.12 to 0.3)	9.56×10^{-12}	-1.956143	0.14 (0.09 to 0.22)	2.18×10^{-18}
Soap	-1.921207	0.15 (0.09 to 0.23)	1.20×10^{-16}	-2.144416	0.12 (0.08 to 0.18)	2.56×10^{-23}
Cinnamon	-1.594497	0.2 (0.13 to 0.32)	2.77×10^{-12}	-1.80051	0.17 (0.11 to 0.25)	6.17×10^{-18}
Watermelon	-1.156884	0.31 (0.19 to 0.52)	7.44×10^{-6}	-1.672423	0.19 (0.12 to 0.30)	4.71×10^{-13}
Lemon	-1.467902	0.23 (0.15 to 0.35)	1.73×10^{-11}	-1.581007	0.21 (0.14 to 0.31)	5.87×10^{-15}
Natural gas	-1.307766	0.27 (0.16 to 0.46)	1.75×10^{-6}	-1.602784	0.20 (0.12 to 0.33)	1.91×10^{-10}
Paint thinner	0.8308298	0.44 (0.28 to 0.67)	1.88×10^{-4}			
Rose	0.8286838	0.44 (0.27 to 0.70)	6.57×10^{-4}			
Banana	0.7702489	0.46 (0.29 to 0.73)	0.001			
Pineapple	0.6421734	0.53 (0.32 to 0.86)	0.011			
Chocolate	0.6277841	1.87 (1.10 to 3.20)	0.021			
Motor oil	0.5198073	0.59 (0.40 to 0.93)	0.023			
Menthol	0.6861642	0.50 (0.28 to 0.90)	0.021			
Mint	0.5477109	1.73 (1.03 to 2.89)	0.037			

Supplementary Figure 1: Distribution of likelihood ratios among Parkinson's disease (PD) cases and controls (A), and the observed detection rate according to false-positive rate (receiver operating characteristic curve; B) for the logistic regression model based on the four odours that discriminated most between those with and without hyposmia from previous work, plus a further two odours that most discriminated between PD and controls. [Joseph et al 2019, Auger et al 2020]:

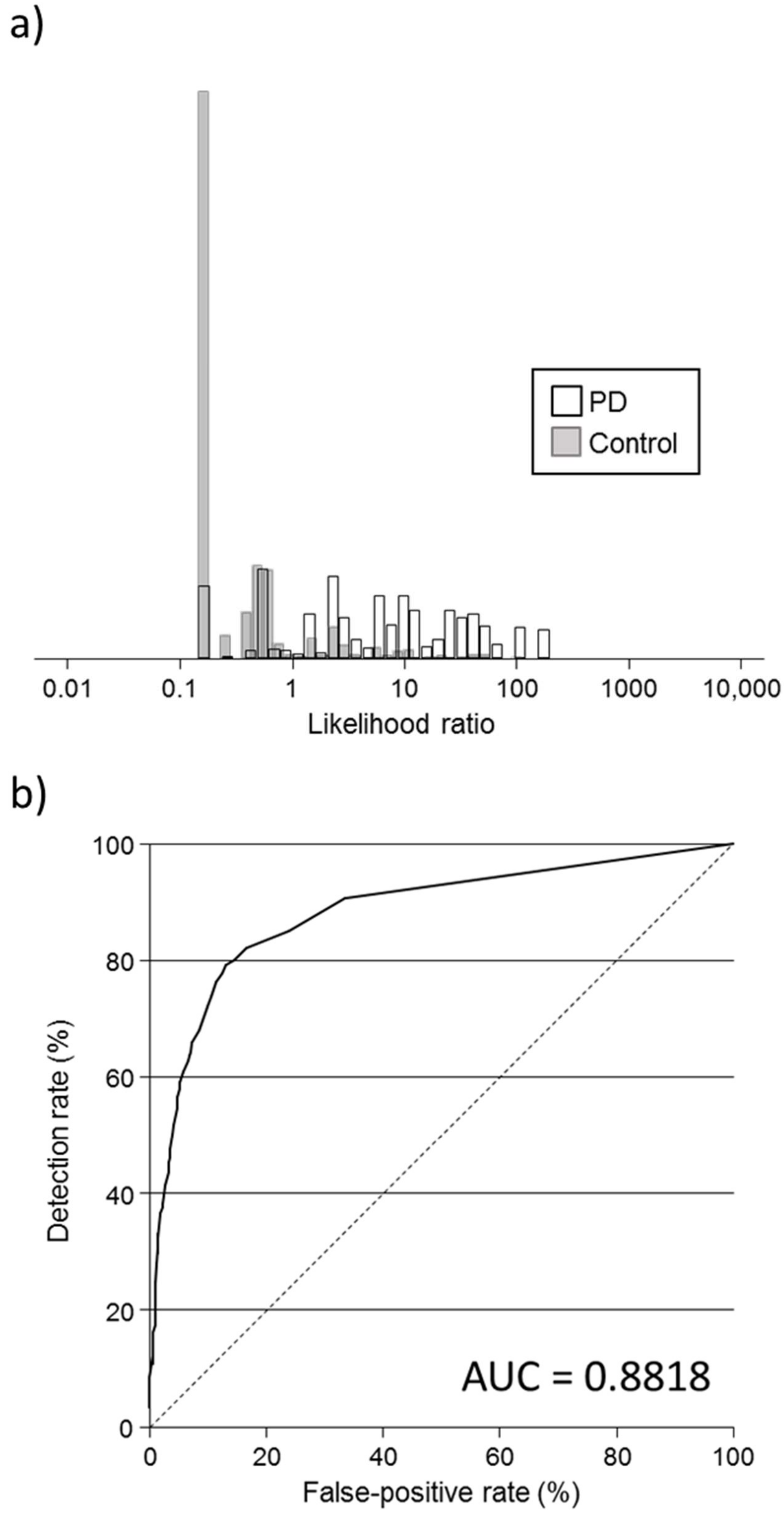

AUC; area under the receiver operating characteristic curve

Supplementary Figure 2: Distributions of delta UPSIT scores (observed score minus expected score according to age and gender) among Parkinson's disease (PD) patients and controls (A), the likelihood ratio according to delta UPSIT scores with dashed lines indicate where truncation is applied (B), the observed detection rate according to false-positive rate (receiver operating characteristic curve) for delta UPSIT values (C)

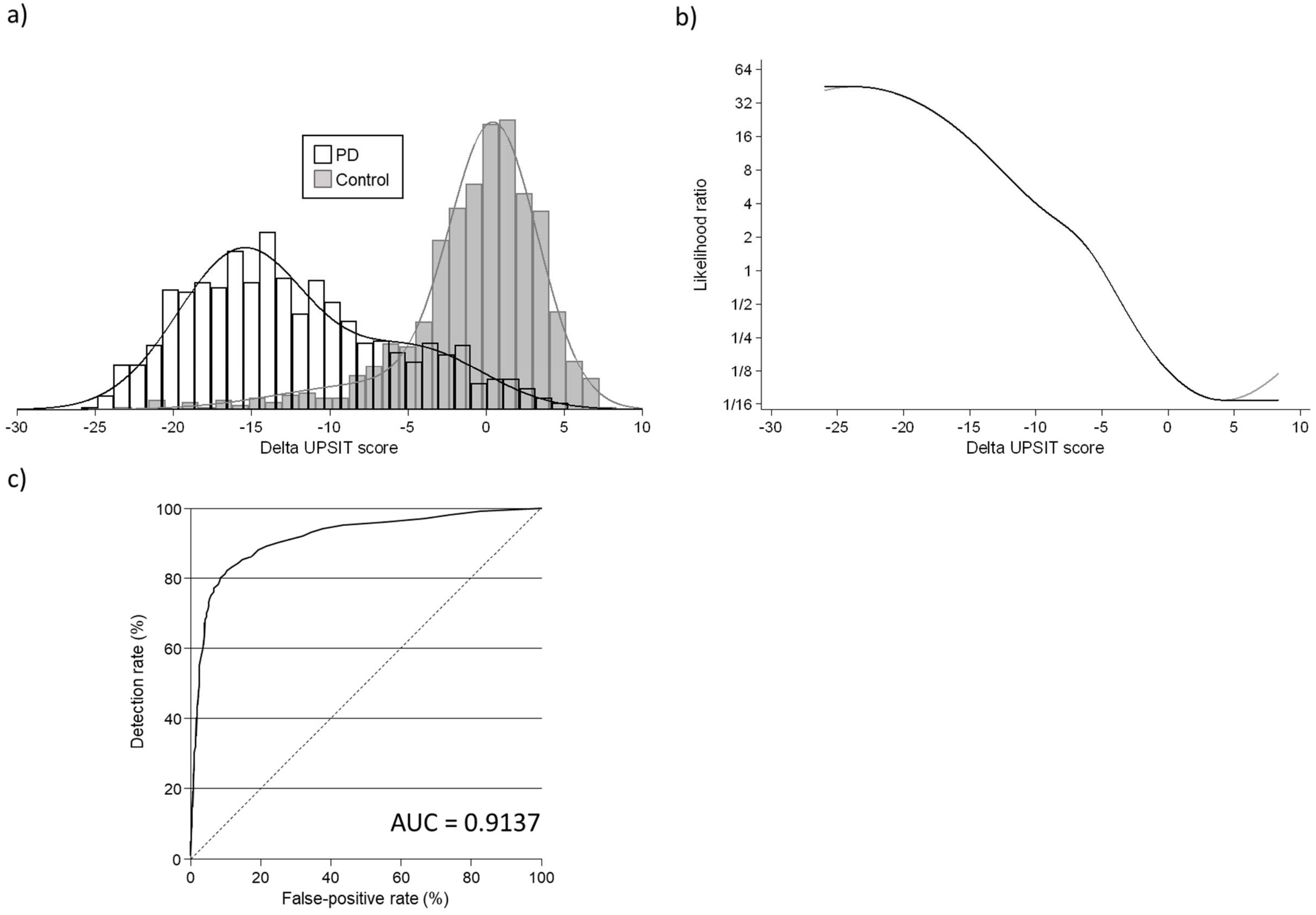

AUC; area under the receiver operating characteristic curve

Supplementary Figure 3: Probability plots of delta kinesia scores (KS) (A) and akinesia time (AT) multiple of the median (MoM) values (B) among Parkinson's disease (PD) patients and controls.

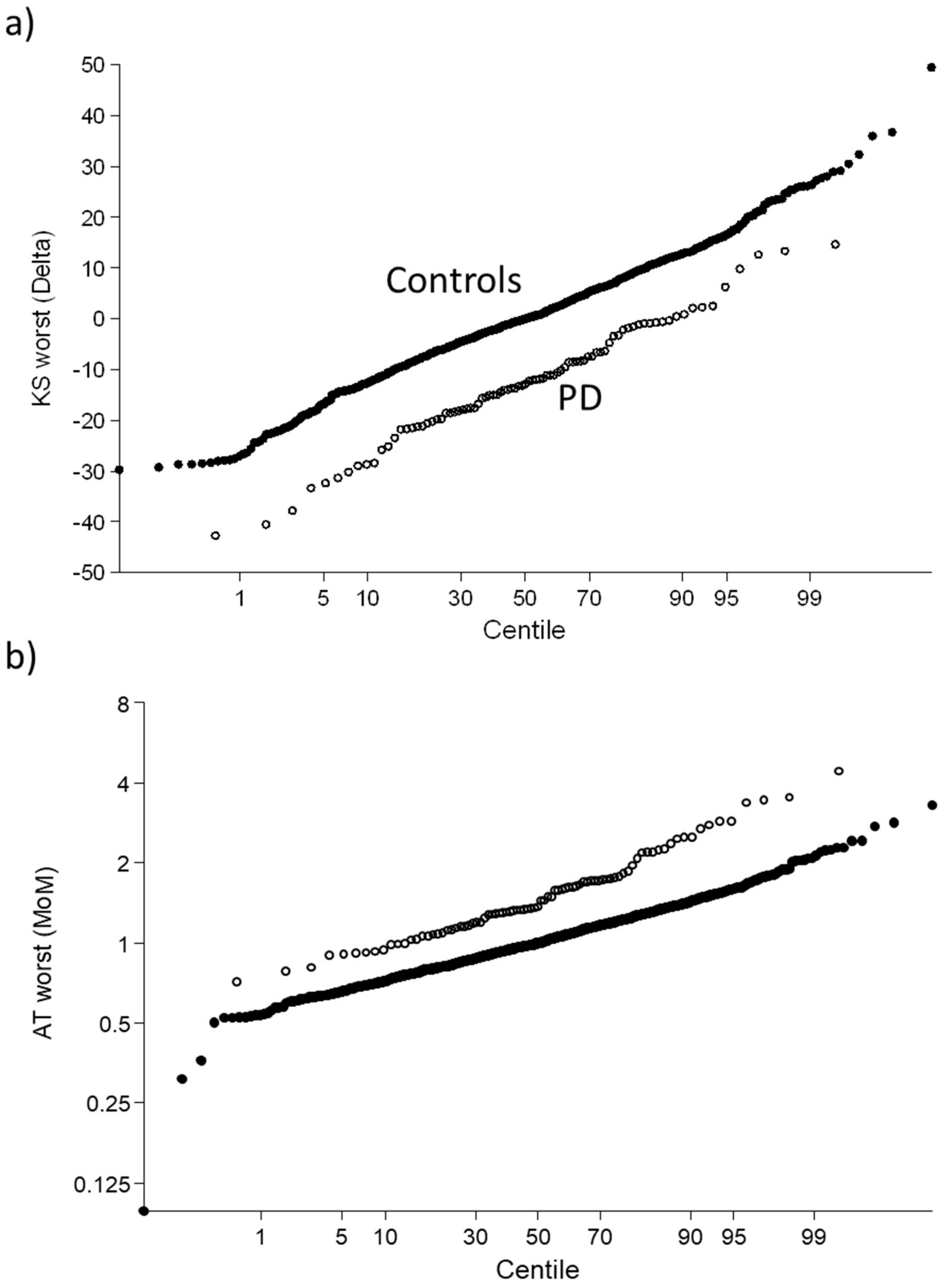

Supplement: Supplementary file 1 — Supplementary Information [file 41531_2021_226_MOESM1_ESM.pdf]
