# Supplementary material for: Optimising classification of Parkinson’s disease based on motor, olfactory, neuropsychiatric and sleep features
Source: NPJ Parkinsons Dis. 2021 Sep 24;7:87. doi: 10.1038/s41531-021-00226-2 (PMC8463675; doi:10.1038/s41531-021-00226-2)
Supplement: Supplementary file 2 — Reporting Summary [file 41531_2021_226_MOESM2_ESM.pdf]

nature portfolio

Corresponding author(s): Jonathan Bestwick

Last updated by author(s): Aug 5, 2021

Reporting Summary

Nature Portfolio wishes to improve the reproducibility of the work that we publish. This form provides structure for consistency and transparency in reporting. For further information on Nature Portfolio policies, see our Editorial Policies and the Editorial Policy Checklist.

Statistics

For all statistical analyses, confirm that the following items are present in the figure legend, table legend, main text, or Methods section.

n/a	Confirmed	
☐	☒	The exact sample size (n) for each experimental group/condition, given as a discrete number and unit of measurement
☐	☒	A statement on whether measurements were taken from distinct samples or whether the same sample was measured repeatedly
☐	☒	The statistical test(s) used AND whether they are one- or two-sided<br>*Only common tests should be described solely by name; describe more complex techniques in the Methods section.*
☐	☒	A description of all covariates tested
☐	☒	A description of any assumptions or corrections, such as tests of normality and adjustment for multiple comparisons
☐	☒	A full description of the statistical parameters including central tendency (e.g. means) or other basic estimates (e.g. regression coefficient) AND variation (e.g. standard deviation) or associated estimates of uncertainty (e.g. confidence intervals)
☐	☒	For null hypothesis testing, the test statistic (e.g. F, t, r) with confidence intervals, effect sizes, degrees of freedom and P value noted<br>*Give P values as exact values whenever suitable.*
☒	☐	For Bayesian analysis, information on the choice of priors and Markov chain Monte Carlo settings
☒	☐	For hierarchical and complex designs, identification of the appropriate level for tests and full reporting of outcomes
☐	☒	Estimates of effect sizes (e.g. Cohen's d, Pearson's r), indicating how they were calculated

Our web collection on statistics for biologists contains articles on many of the points above.

Software and code

Policy information about availability of computer code

Data collection	*Provide a description of all commercial, open source and custom code used to collect the data in this study, specifying the version used OR state that no software was used.*
Data analysis	Stata version 15

For manuscripts utilizing custom algorithms or software that are central to the research but not yet described in published literature, software must be made available to editors and reviewers. We strongly encourage code deposition in a community repository (e.g. GitHub). See the Nature Portfolio guidelines for submitting code & software for further information.

Data

Policy information about availability of data

All manuscripts must include a data availability statement. This statement should provide the following information, where applicable:

- Accession codes, unique identifiers, or web links for publicly available datasets
- A description of any restrictions on data availability
- For clinical datasets or third party data, please ensure that the statement adheres to our policy

Applications for PREDICT-PD data are reviewed by our steering committee
Applications for Tracking Parkinson's data access are reviewed by the Data & Biosample Access committee

Field-specific reporting

Please select the one below that is the best fit for your research. If you are not sure, read the appropriate sections before making your selection.

☒ Life sciences ☐ Behavioural & social sciences ☐ Ecological, evolutionary & environmental sciences

For a reference copy of the document with all sections, see nature.com/documents/nr-reporting-summary-flat.pdf

Life sciences study design

All studies must disclose on these points even when the disclosure is negative.

Sample size	Data were taken from other studies that were powered according to their endpoints
Data exclusions	Data restricted to 60-80 years of age at baseline for consistency between datasets
Replication	Internal validation was performed using bootstrapping and validated estimates were similar to those observed in the original data
Randomization	Not applicable - this is not an interventional study
Blinding	Not applicable - this is not an interventional study

Reporting for specific materials, systems and methods

We require information from authors about some types of materials, experimental systems and methods used in many studies. Here, indicate whether each material, system or method listed is relevant to your study. If you are not sure if a list item applies to your research, read the appropriate section before selecting a response.

Materials & experimental systems

n/a	Involved in the study	
☒	☐	Antibodies
☒	☐	Eukaryotic cell lines
☒	☐	Palaeontology and archaeology
☒	☐	Animals and other organisms
☐	☒	Human research participants
☒	☐	Clinical data
☒	☐	Dual use research of concern

Methods

n/a	Involved in the study	
☒	☐	ChIP-seq
☒	☐	Flow cytometry
☒	☐	MRI-based neuroimaging

Human research participants

Policy information about studies involving human research participants

Population characteristics	Controls: mean age 67 years, 67% female<br>Cases: mean age 69 years, 47% female
Recruitment	PREDICT-PD participants aged 60-80 years without PD were recruited by self-referral. Patients attending specialist clinics in the United Kingdom with recent onset (<3.5 years) and young onset (diagnosed<50 years of age) PD were enrolled in the Tracking Parkinson's study
Ethics oversight	The PREDICT-PD study was approved by Central London Reserch Committee 3 (reference number 10/H0716/85). 72 sites in the UK providing secondary care treatment for PD patients as part of the UK National Health Service (NHS) (and in selected sites, their linked academic institutions) are participating in the Tracking Parkinson's study, with multicentre ethics committee and local research and development department approvals.

Note that full information on the approval of the study protocol must also be provided in the manuscript.
